# Influence of physical activity on loneliness and depression among the older adults in Nigeria

**DOI:** 10.1038/s41598-024-77263-3

**Published:** 2024-11-05

**Authors:** Joel O. Faronbi, Mariam O. Ojewale, Grace O. Faronbi, Olagbegi M. Oladapo

**Affiliations:** 1 Academy of Nursing, Department of Health and Care Profession, University of Exeter, Exeter, UK; 2 Department of Nursing Science, College of Health Sciences, Obafemi Awolowo University, Ile–Ife, Nigeria; 3 Department of Nursing Education, University of the Witwatersrand, Johannesburg, South Africa; 4 Division of Physiotherapy, School of Health Sciences, University of KwaZulu-Natal, Durban, South Africa

**Keywords:** Physical activity, Loneliness, Depression, Older adult, Psychology, Diseases, Medical research

## Abstract

This study aims to determine the predictive influence of physical activities (PA) and evaluate the interactive effect of PA and loneliness on depression among older adults in Nigeria. Data was collected from 369 randomly selected older adults and analysed using descriptive statistics, chi-square test and logistic regression models. The result showed that that the predictors of depression were loneliness (OR = 4.59; *p* < 0.001), PA *High* (OR = 2.51; *p* = 0.008), Age *80–89*(OR = 9.63; *p* < 0.001), education: *Secondary* (OR = 2.04; 0.049), religion (OR **=** 0.30; p 0 < 0.001) and living arrangement: *Family* (OR = 1.87; *p* = 0.037. The study further showed that there is an interactive effect of PA to reduce the odds of loneliness on depression (OR reduced from 4.59 to 3.40). The study concluded that the predictors of depression in this population are loneliness, physical activity, age, education, and religion while depression and the living arrangements are the predictors of loneliness among older adults.

## Introduction

Depression in older people is currently and might continue to be a public health problem, as it concerns a growing age group. There is a high rate of comorbidity with physical illness and impaired functioning^10^. Depression has an impact on older adults, and it is a significant contributor to morbidity around the world and among older Africans^23^. Prevalence estimates among those aged 50 and older suggest that 3.2 per cent of Ghanaians and 2.8 per cent of South Africans had depression, with prevalence increasing with age in Ghana^23^. Additionally, among older adults in specific communities in Nigeria, a 12.1 per cent prevalence of depression has been reported^37^. The recognition of depression as part of healthcare for the older adult is crucial because of the impact of depression on other aspects of that individual health, even if the older adult person has only low mood or sadness or minor depression^46^. Depression in older people often goes undetected by physicians^10^, but it has severe consequences on physical health, functioning and overall quality of life^16^.

Loneliness is a subjective experience or perception of being isolated, and it is an essential concern for the welfare of older people. It also has health implications^41^. Loneliness can be described as the discrepancy between a person’s desired and actual relationship^10^. Research has linked loneliness to higher risks for a variety of physical and mental conditions: high blood pressure, heart disease, obesity, a weakened immune system, anxiety, depression, cognitive decline, Alzheimer’s disease and even death^35^. Loneliness among older people is widespread; for instance, 20–34% of older people in China, Europe, Latin America, and the United States of America are lonely^52^. Loneliness increases as individuals aging; 43.2% of individuals 65 or older reported feeling lonely at least some time^29^. The links between loneliness and its harmful physical and mental health consequences are widely reported^28^ and include increased risk of mortality^28^. Despite the range of services and activities to alleviate loneliness, the prevalence in community-dwelling older people has remained constant over the last few decades^28^.

WHO defines physical activity as any bodily movement produced by skeletal muscles that requires energy expenditure^50^. Physical activity refers to all movement, including during leisure time, for transport to and from places or as part of a person’s work^53^. It includes walking and cycling, active play, work-related activities, and active recreation such as gym work, dancing, gardening, and sports^53^. Because exercise capacity (physical fitness) among older adults tends to decrease as they age, they need an age-appropriate physical activity plan that meets their requirements^51^. Risk factors for physical inactivity include the presence of loneliness, depression, hypertension, heart disease, osteoporosis, colon and breast cancer, obesity, adult-onset diabetes, and anxiety^29,49^. A significant correlation was identified between physical activity, depression and anxiety^47^.

Prevention of depression in older adults could offer possibilities to avoid the sudden decline in overall health status. Physical activity contributes to the reduction of psychological distress among older adults because it promotes psychosocial interaction, is an effective factor self-esteem, helps in the maintenance and improvement of cognitive functions, and reduces the frequency of relapse of depression and anxiety^47^.

With the predicted increase of older adults’ population worldwide, the burden of loneliness, depression, and physical inactivity may also worsen over time if unattended^29^. Therefore, the objectives of the study are to determine predisposing factors for depression and loneliness, determine the influence of physical activity on loneliness and depression and evaluate the interactive effect of physical activity and loneliness on depression among the older adults in Ife North Local Government.

## Methodology

This cross-sectional research was carried out in Ife North Local Government Area (LGA). Ife North is a local government area in Osun State, Nigeria. Its headquarters is in the town of Ipetumodu in the North of the area at 7^o^31’00’’N 4^o^27’00’’E. According to the official 2006 census^36^, it has an area of 889 km2 and about 153,694 inhabitants. The LGA includes other districts such as Edunabon, Moro, Yakoyo, Ashipa, Akinlalu, Famia, and Oyere.

The target population included all adults 60 years of age and above, residing in the LGA for at least one year before the study. This was to ensure that data were collected from only regular inhabitants of the town, ensuring the enrolment of stable residents. According to the 2006 population census, 9,390 individuals aged 60 years and above reside within the LGA^36^. The inclusion criteria for the study are individuals 60 years and above who have been residents in the area for more than one year, while those with any cognitive impairment or not able to give informed consent were excluded from the study.

The sample size was derived using the formula n = Z^2^pq/d^2^^34^. Where n represents the sample size, p represents the prevalence of depression among older adults, which some Nigerian authors documented as 42.5%^45^ or 44.7% ^4^. Therefore, we used 45% of the population for the sample size calculation, with a 10% non-response rate, giving a sample size of 380.

A multi-stage sampling technique was used to select participants. Three major towns, Ipetumodu, Edunabon, and Moro, were randomly selected from the seven major towns of Ife North Local Government. Each of these towns, or communities, was divided further into enumeration areas (EAs) for demographic purposes; Ipetumodu has 800 EAs, Edunabon has 340 EAs, and Moro has 130 EAs, according to the Ife North Local Government Local Government Office of the National Population Commission. Ten per cent of EAs were randomly selected from each town in proportion to the number of EAs in each town. This translated to 80 EAs for Ipetumodu, 34 EAs for Edunabon, and 13 EAs for Moro. Thereafter, households were randomly selected from each EA; where the selected household had at least one eligible respondent. Three households were randomly selected in each of the EA. Inclusion criteria for the study were older adults (aged 60 years and over), residents in the study area and cognitively intact. In the case of more than one eligible participant in the dwelling, we enrolled only one using the lottery method. Data were collected after permission was obtained from the traditional rulers of each community, and informed consent was obtained from each participant. However, 369 complete questionnaires were deemed fit for data analysis, yielding a response rate of 97%.

### Instruments for data collection

The data collection instrument was a questionnaire consisting of four parts: the socio-demographic characteristics of the subjects, the Geriatric Depression Scale, the UCLA Loneliness Scale (ULS-3) and PA. The Geriatric Depression Scale (GDS) was used to assess depression. The 15-item instrument developed by^43^ has been tested and used extensively with the older Nigerian population^2^. A scoring grid accompanies the GDS. One point is given for each respondent’s answer that matches those on the grid. Each question is scored as either 0 or 1 point. Scores of 0–4 are considered normal; 5–8 indicate mild depression; 9–11 indicate moderate depression; and 12–15 indicate severe depression.

The University of California Loneliness Scale (UCLA) was used to assess loneliness^24^ and has high internal consistency (coefficient alpha = .96) and a test-retest correlation over two months of .73 (Avci, 2018). In order to dichotomise loneliness, a score of greater than 3 (those who answer ‘some of the time’ or ‘often’ to any item) is used to assess the presence of loneliness^9^. A higher score demonstrates a higher level of loneliness. We assessed the physical activity of the respondents using the International Physical Activity Questionnaire (IPAQ). The IPAQ assesses physical activity undertaken across a comprehensive set of domains, including leisure-time physical activity, domestic and gardening (yard) activities, work-related physical activity, and transport-related physical activity^7^. The IPAQ uses two scoring protocols: (i) continuous score and (ii) category score, and the Cronbach alpha for the IPAQ was established in a previous study as 0.714 (Ayvat et al. 2017).

## Ethics

All methods were carried out in accordance with relevant guidelines and regulations, and the all-experimental protocols were approved by the Research Ethic Committee of Obafemi Awolowo University Teaching Hospitals Complex, Ile-Ife, Nigeria (ERC/2021/01/10) and Osun State Ministry of Health OSHREC/PRS/569T/165. In addition, all the participants gave informed consent before participation in the research, and they were assured of the confidentiality of any information provided. All the participants gave informed consent regarding publishing of anonymous data collected for the study.

## Method of data analysis

Data was analysed descriptively (frequency, percentage, mean, standard deviation) to describe the socio-demographic characteristics and other variables in the study. Similarly, correlation analysis was conducted to assess the relationship between the different variables of older adults. The chi-square test was used to evaluate the differences in sample characteristics; logistic regression analysis was used to assess the association between physical activity, loneliness, age, gender, marital status, occupation, level of education, ethnicity, religion, living arrangement, and financial status (independent variables), and depression and loneliness (outcome variables). All analysis was conducted using Stata 18 statistical software^44^.

## Results

The result shows the age ranges from 60 to 99 years with a mean of 72.30+/- 8.67, and almost half (44.4%) of the respondents were between 70 and 79 years of age. Majority (63.2%) of the respondents were married and more than half (58.3%) were female. An overwhelming majority (85.1%) were Yorubas. More than two-thirds (68.0%) of the respondents were Christians, and more than one-third (36.0%) had no formal education. Less than half (45.0%) of the respondents live with their children or spouse. More than half (50.7%) of the respondents are self-employed, and an overwhelming (83.5%) were living with one form of illness or the other (see Table 1).

**Table 1:** Socio-demographic characteristics and other variables of the respondents.

Variable	Frequency (*n*)	Percent (%)
**Age**		
60–69	136	36.9
70–79	164	44.4
80–89	52	14.1
> 90	17	4.6
**Sex**		
Male	154	41.7
Female	215	58.3
**Ethnicity**		
Yoruba	314	85.1
Igbo	29	7.9
Hausa	26	7.0
**Education**		
No formal education	133	36.0
Primary	93	25.2
Secondary	78	21.1
Tertiary	65	17.6
**Religion**		
Christian	251	68.0
Islam	118	32.0
**Marital**		
Married	239	64.8
Divorce/separated	130	35.2
**Occupation**		
Self-employed	187	50.7
Civil servant	52	14.1
Retiree	130	35.2
**Morbidity**		
No	61	16.5
Yes	308	83.5
**Whom do you live with**		
Living with spouse	203	55.0
Living Alone	166	45.0
**Loneliness**		
Not lonely	167	45.3
Lonely	202	54.7
**Physical Activity**		
Low	114	30.9
Moderate	52	14.1
High	203	55.0
**Depression**		
Not depressed	142	38.48
Depressed	227	61.52

**Fig. 1:**
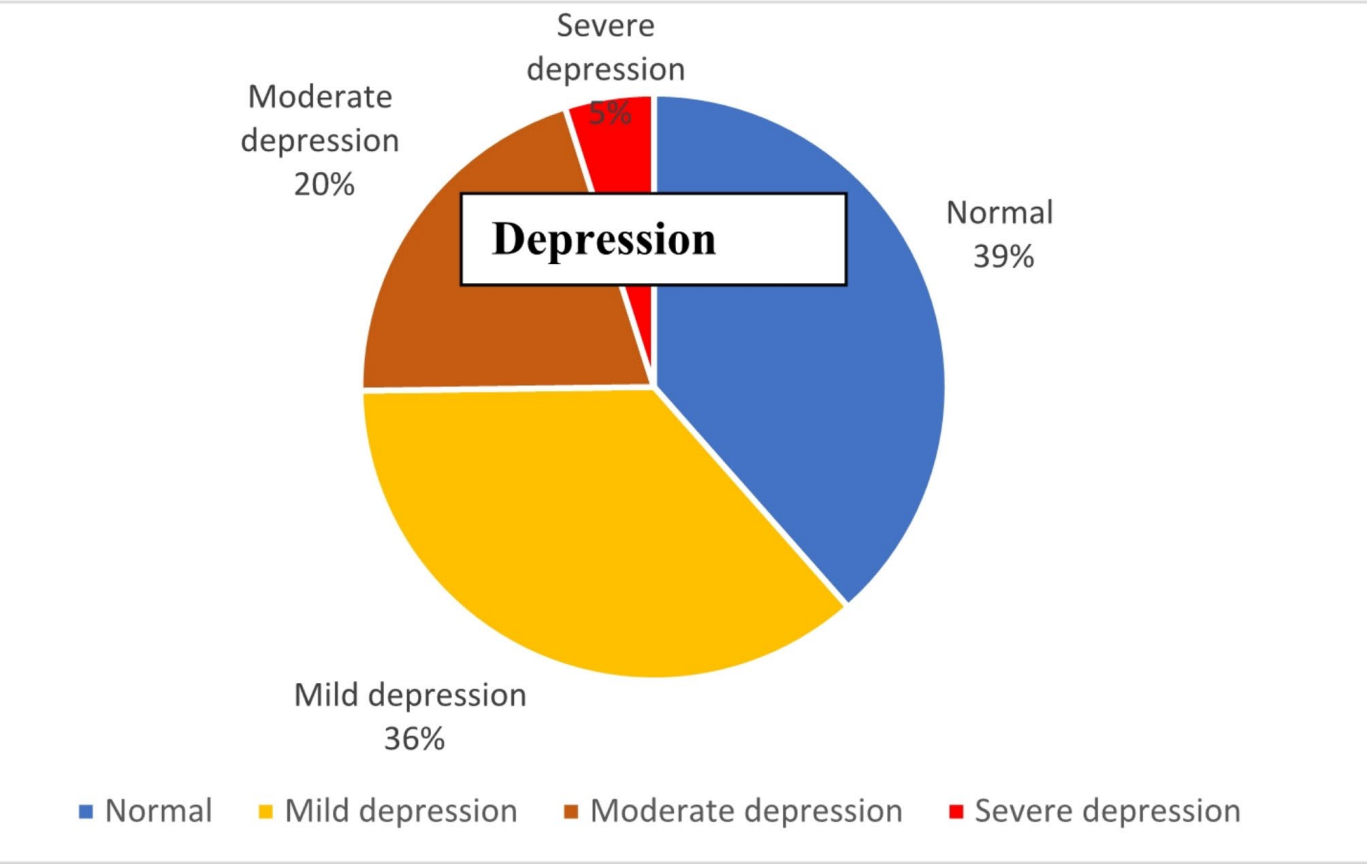
Level of depression among older adult.

**Fig. 2:**
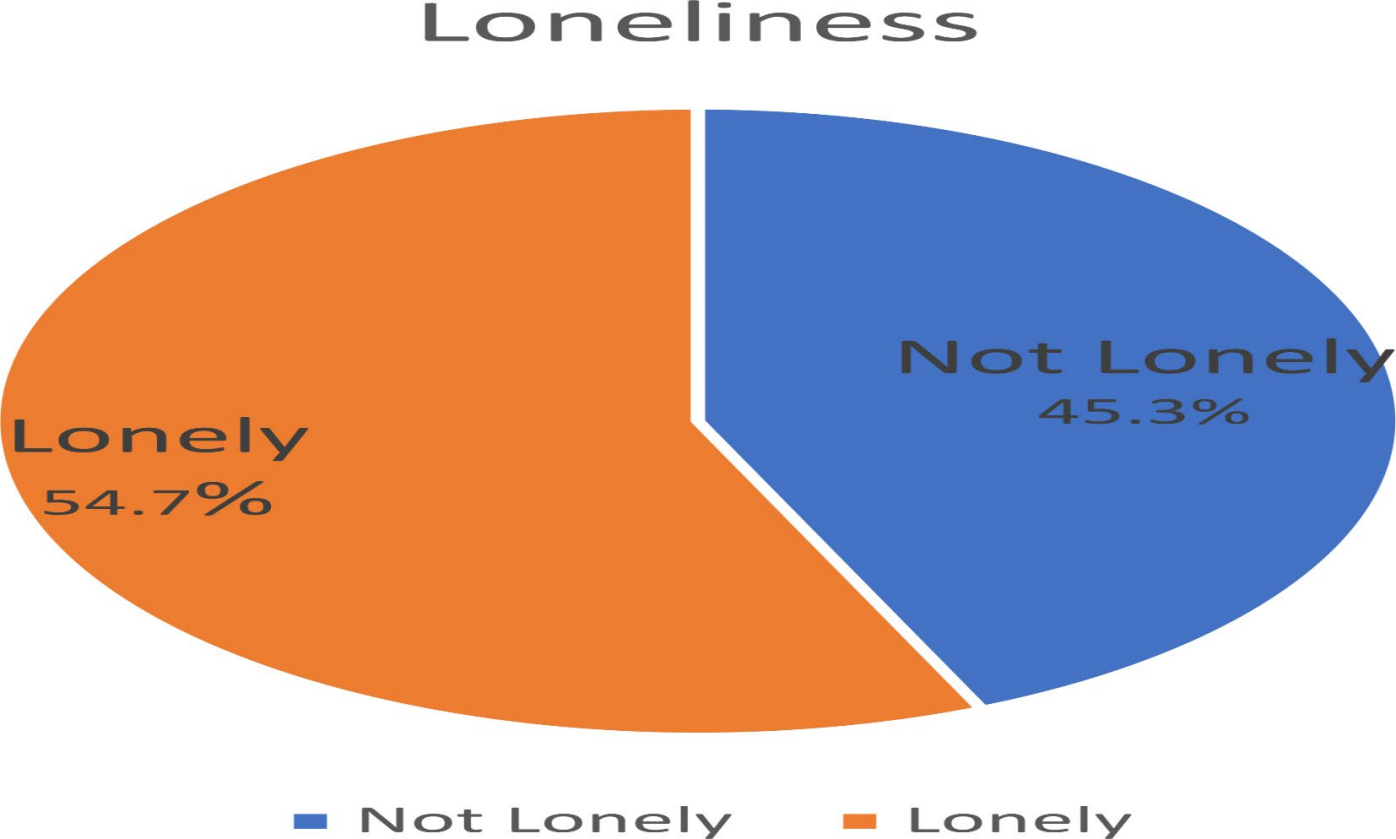
Loneliness among the older adults.

Figure 1 shows the summary of the level of depression among older adults in this setting. A large proportion (61.5%) of respondents had various levels of depression ranging from mild (35.5%), moderate (21.1%) and severe depression (4.9%). Similarly, Fig. 2 shows the level of loneliness among older adults in this setting with more than half (53.7%) being lonely.

Table 2 presents the results of bivariate analysis of the associations of independent variables (Age, Sex, Ethnicity, Education, Religion, Marital, Occupation, Morbidity, living arrangement, Loneliness, and Physical Activity) and depression (dependent variable) among older adults. The result showed that age (*p* = 0.018), education (*p* < 0.001), religion (*p* < 0.001), living arrangement (*p* = 0.001), physical activity (*p* = 0.019), and loneliness (*p* < 0.001) were significantly associated with depression.

**Table 2:** Association of physical activity and depression among the older adults.

Variable	Not Depressed (*n* = 142)	Depressed (*n* = 227)	Total	χ ^2^	*P*
**Age**					
60–69	55	81	136	10.04	0.018
70–79	71	93	164		
80–89	10	42	52		
> 90	6	11	17		
**Sex**					
Male	52	102	154	2.48	0.115
Female	90	125	215		
**Education**					
No formal education	71	62	133	55.17	< 0.001
Primary	45	48	93		
Secondary	25	53	78		
Tertiary	1	64	65		
**Religion**					
Christian	75	176	251	24.53	< 0.001
Islam	67	51	118		
**Marital**					
Married	91	148	239	0.05	0.827
Living alone	51	79	130		
**Occupation**					
Self-employed	71	116	187	0.24	0.887
Civil servant	22	31	53		
Retiree	49	80	129		
**Illness**	0	1			
No	20	41	61	1.00	0.317
Yes	122	186	308		
**Living arrangement**					
Caregiver	82	121	203	0.70	0.404
Family	60	106	166		
**Physical Activity**					
Low	56	58	114	7.91	0.019
Moderate	18	34	52		
High	68	135	203		
**Loneliness**					
Not lonely	96	71	167	46.53	< 0.001
Lonely	46	156	202		

A logistic regression analysis was run to identify independent predictors (loneliness, physical activity, morbidity, education, religion, gender, age, marital status, occupation, financial status, and living arrangement) for depression. The first model (without interaction of loneliness and PA) showed that Loneliness (OR = 4.59; *p* < 0.001), Physical activity: *High* (OR = 2.51; *p* = 0.008), Age *80–89* (OR = 9.63; *p* < 0.001), Education: *Secondary* (OR = 2.04; 0.049), *Tertiary* (OR = 90.29; *p* < 0.001), Religion (OR **=** 0.30; p 0 < 0.001) and Living arrangement: *Family* (OR = 1.87; *p* = 0.037 were significantly associated with depression (See Table 3). The second model contained the interaction between loneliness and PA on depression. The profound result in the second model is the reduction of the odds ratio of loneliness from OR = 4.59 to OR 3.40. Similarly, a logistic regression analysis revealed that depression and living arrangements of older adults are the predictors of loneliness among them (See Table 4).

**Table 3:** Logistic regression analysis predicting variables associated with depression among the respondents.

Model 1, Logistic regression without interaction					Model 2, Logistic regression with interaction				
Depression	OR	*P* > z	[95% CI]		Depression	OR	*P* > z	[95% CI]	
**Loneliness**	4.59	**< 0.001**	2.64	7.98	**Loneliness**	3.4	**0.009**	1.36	8.5
**Physical activity**					**Physical activity**				
Low	1				Low	1			
Moderate	1.83	0.213	0.71	4.73	Moderate	1.35	0.639	0.39	4.69
High	2.51	**0.008**	1.27	4.96	High	2.03	0.12	0.83	4.94
					**Loneliness## Physical activity**				
					Lonely # moderate	1.94	0.483	0.3	12.35
					Lonely #High	1.51	0.493	0.47	4.85
**Age**					**Age**				
60–69	1				60–69	1			
70–79	1.55	0.216	0.77	3.11	70–79	1.56	0.216	0.77	3.16
80–89	9.63	**< 0.001**	3.01	30.77	80–89	9.74	**< 0.001**	3.04	31.2
> 90	2.19	0.31	0.48	9.94	> 90	2.3	0.279	0.51	10.36
**Education**					**Education**				
No formal education	1				No formal education	1			
Primary	1.23	0.534	0.64	2.36	Primary	1.24	0.514	0.65	2.39
Secondary	2.04	**0.049**	1	4.17	Secondary	2.02	0.054	0.99	4.12
Tertiary	90.29	**< 0.001**	11.21	727.33	Tertiary	91.25	**< 0.001**	11.31	735.98
**Sex**	0.61	0.158	0.3	1.21	**Sex**	0.62	0.173	0.31	1.24
**Religion**	0.3	**< 0.001**	0.16	0.54	**Religion**	0.3	**< 0.001**	0.16	0.54
**Living arrangement**	1.87	**0.037**	1.04	3.38	**Living arrangement**	1.9	**0.034**	1.05	3.46
**Occupation**					**Occupation**				
Self-employed	1				Self-employed	1			
Civil servant	0.76	0.498	0.34	1.69	Civil servant	0.75	0.486	0.34	1.68
Retiree	0.56	0.134	0.26	1.19	Retiree	0.57	0.141	0.27	1.21
**Marital status**	0.98	0.953	0.52	1.85	**Marital status**	0.98	0.942	0.52	1.85
**Illness**	0.83	0.633	0.39	1.78	**Illness**	0.82	0.615	0.38	1.77
*n* = 369, χ ^2^ (16) = 157.02, *p* < 0.001; R^2^ = 0.319					*n* = 369, χ ^2^ (18) = 157.73, *p* < 0.001; R^2^ = 0.321				

**Table 4:** Logistic regression analysis predicting variables associated with loneliness among the respondents model 3.

Loneliness	OR	*P* > z	[95% CI]	
**Depression**	4.53	**< 0.001**	2.64	7.78
**Living arrangement**	1.83	**0.018**	1.11	3.02
**Physical activity**				
moderate	0.56	0.155	0.25	1.25
High	0.83	0.531	0.45	1.50
**Age**				
60–69	1			
70–79	0.96	0.887	0.53	1.72
80–89	1.71	0.274	0.65	4.49
> 90	2.54	0.19	0.63	10.22
**Sex**	0.84	0.547	0.47	1.49
**Religion**	0.87	0.616	0.51	1.50
**Marital**	0.68	0.17	0.39	1.18
**Occupation**				
Self-employed	1			
Civil servant	0.58	0.114	0.29	1.14
Retiree	0.61	0.136	0.32	1.17
**Illness**	1.30	0.407	0.70	2.43
**Education**				
No formal education	1			
Primary	1.01	0.976	0.55	1.86
Secondary	0.94	0.861	0.49	1.82
Tertiary	1.30	0.487	0.62	2.73
	*n* = 369, χ ^2^(16) = 67.37, p = < 0.001 R^2^ = 0.1326			

Significant values are in bold.

## Discussion of findings

The findings of this current study revealed that a large proportion (61.5%) of respondents had various levels of depression ranging from mild (35.5%), moderate (21.1%) and severe depression (4.9%). Overall prevalence (61.5%) is higher than the prevalence of depression reported in previous Nigerian studies 48.5%^26^, 45.5%^2^, and 12.1%^38^. This higher prevalence might be attributed to many reasons, including the high prevalence of loneliness, advanced age and associated chronic illness among the older adults in this population. Furthermore, the 5% of older adults with severe depression in this study may not be ignored and would need immediate intervention. This might include designing interventions that promote physical and social interactions, and this will likely go a long way in reducing loneliness and depression among older adults.

The findings of this current study revealed that more than half of the respondents are lonely and that there is a significant relationship between the level of depression and loneliness among older adults. Although most of these senior people live among their relatives, some might be lonely because it is possible to live in lack amid plenty. They may lack companionship and feel isolated from others. This may happen because of lack of connection and not just living in among people^3^. This finding is higher than that obtained from a previous study among older adult respondents in Nigeria^25^. Also, differences in physical activity may contribute to the increased risk of ill-health and poor wellbeing associated with isolation^41^. The result agrees with and recognises loneliness as a strong correlate of depressive symptoms; loneliness is one of the strongest predictors of depression^22^.

Also, findings show that loneliness is associated with depression. This is supported by Igbokwe^25^ in their study, which stated that lonely respondents are twice as likely to be depressed compared to non-lonely counterparts. The result is also in agreement with other studies that showed that loneliness had a moderately significant effect on depression^11,12,48^.

The interactive effect of PA on loneliness to decrease the risk of depression is also noted in this study. With PA, there is a decrease odd of loneliness on depression from (OR = 4.67 to 3.45). Another important finding of this study is that only those with high PA is significant. This also suggests the need to promote a sustained age-appropriate PA among older adults. Most older adults engage in some form of PA in the course of their daily activities. One important point among this population is that most of the older adults still engage in economic activities even up till latter age. Previous studies^14,18,31^ established that a large number of older adults engaged in one form of economic activity or the other that being physically active has a positive impact on the long-term income and of maintaining or enhancing self-esteem^19^. suggest that the capacity of older adults must be better developed and utilized in activities that make economic contributions to society.

Furthermore, findings from this study showed that physical activity provides a protective effect against depression among older adults. Physically active older adults would be able to move around, socialise, participate in religious activities, and engage in economically productive activities, thereby having a multiplier health benefit. However, as some of them continue to grow older, their level of activities continues to decline and hence this negatively affects their muscle strength, endurance, and body structure^15,33^, and thereby making them to be physically inactive and this might have a negative effect on their wellbeing. The relatively high levels of physical activities demonstrated by older people should be encouraged to provide a sustained protective effect against loneliness and depression among the respondents. Conscientious and vigorous efforts should be put in place because the tendencies for physical activities tend to decline with increasing age^39^. This suggests that designing an intervention package that promotes physical activity among older adults might likely be of great benefit in indirectly addressing isolation, loneliness, and depression^27^. Some of the activities that respondents participate in include walking, jogging, table tennis, playing (age-appropriate) and watching football^6,40^.

Continuous work engagement in economic activities is important to promote social interaction among the older adults. Some may not even be doing the work for economic reasons but to stay away from boredom. Work engagement provides opportunities to socialise and interact, thereby reducing tendencies for isolation, loneliness, and depression. Prolonged work participation is already visible in older workers who decide to continue their engagement in work activities beyond the statutory retirement age^42^. Sewdas^42^ further argued that the most important motives for working beyond retirement age were maintaining daily routines and financial benefits. These two reasons might apply to the older adults in this study.

Work enjoyment may be seen as an intrinsic motivator, with work itself being a reward. This might also be associated with the opportunity to maintain relationships and connection and maintain their relationships at work (with co-workers and supervisors) as well as relationships outside of work and maintaining a daily routine that included work^5^. Bratun^5^ argued that health was perceived as a desired outcome of continued work participation. Engagement in work enables older adults to remain active participants in society and to foster social interactions^1,13,21^.

One important finding of this study is the buffer effect of religion against depression among older adults. While all other factors increase the odd of depression, religion on the other hands reduces the odd. A large proportion of the respondents were religious. Nigeria is a predominantly religious country, with most people affiliated with one of the main two prominent religious sects in the country (Christianity and Islam). It is often seen as a taboo for an individual to be without a religion. Most Nigerians spend quite a large proportion of their time in religious gatherings. Apart from the belief that their prayers would be answered by God, it also serves as an opportunity to connect, interact, and socialise. This, on the other hand, shields them against any form of isolation, loneliness, and depression. Most Nigerians, especially older adults, attend religious meetings more than once weekly. The finding regarding religion corroborates^3,8,32^ which found that the participation of the older adult in religious activities was the only form of social engagement associated with a decline in depressive symptoms^30^ further submitted that the community-dwelling older adult population that attended religious services, volunteered, and visited with and talked to friends or neighbours reduced their risk of mortality. Participation in religious gatherings may provide an opportunity for someone to turn to when needing advice, and backing indicates the information and discussions regarding^20^. Zimmer^54^ further argued that there is evidence that religiosity is associated with longer life and better physical and mental health.

Findings from this study also revealed that age is one of the predictors of depression among older adults. Several studies have associated increasing ageing with depression. This might be related to several factors which have been associated with having direct correlation with depression among older adults, including chronic illness, reduced activities, and loneliness^23,37^.

This study also established the influence of education on depression. A good education has been associated with high social, economic status, good standard of living and good quality of life, providing opportunities for access to social amenities and reducing loneliness and depression. Individuals with low socio-economic status (SES) are less likely to use recreational activities compared with those living in high SES areas^17^.

Furthermore, findings from this study established the unique contribution of families in this population. Living arrangements are significantly related to depression and loneliness. Family members might interact with the older person, reducing the risk of loneliness and depression. Although the traditional family structure is being challenged because of the contributions of external migration, older adults with a significant number of family members around them might be less exposed to the risk of depression than those living alone and being attended to by paid caregivers.

### Limitation

The results of the current study should be considered in light of the study’s limitations. First, because of the self-reported nature of all the variables explored in the study, the information was not objectively and clinically validated. This might not rule out the possibility of recall bias. Similarly, some respondents might deny or misrepresent their health conditions while responding to the questionnaire. However, we used verifiable and validated instruments, which strengthened the study, with the possibility of generalising the findings to similar settings.

## Conclusion

The study concludes that depression is highly prevalent among older adults in this population. The result showed that age, education, religion, living arrangements, physical activity, and loneliness are significantly associated with depression. Findings from this study showed that physical activity provides a protective effect against depression among older adults. This then suggests designing an intervention package that promotes physical activity among older adults might likely be of great benefit to address isolation, loneliness, and depression indirectly. This study also revealed the buffering effect of religion against depression among older adults. Participation in religious gatherings may provide a protective effect against depression and prolong longevity with the tendency for religious people to enjoy a longer life and better physical and mental health. The findings of this current study revealed that loneliness is endemic in this population and has a significant relationship with depression.

## Data Availability

“The datasets generated during and/or analysed during the current study are available from the corresponding author on reasonable request.”

## References

[1] Adetunde, C. O., Imhonopi, D., Tayo, O. & Derby, C. N. Socioeconomic adjustment among retired civil servants of Kwara and Lagos States: A Theoretical Analysis. 3rd International Conference on African Development Issues, (2016).

[2] Akosile, C. O. et al. Depression, functional disability and quality of life among Nigerian older adults: Prevalences and relationships. *Arch. Gerontol. Geriatr. ***74**, 39–43. 10.1016/j.archger.2017.08.011 (2018). 28954240 10.1016/j.archger.2017.08.011

[3] Ariyo, O. L. & Faronbi, J. Social Networks and support as an Indicator of Quality of Life: evidence from Elderly Group in Osun State, Nigeria. *J. Med. Sci. Clin. Res. ***10** (4), 8–22 (2022).

[4] Awunor, N. et al. Prevalence and predictors of depression among the elderly in selected rural communities in Delta State, Nigeria. *J. Community Med. Prim. Health Care*. **30** (1), 122–130 (2018).

[5] Bratun, U., Asaba, E. & Zurc, J. Motives of retirement-aged workers and the importance of doing, being, becoming, and belonging: a systematic review of qualitative studies. *J. Occup. Sci. ***30** (3), 420–437. 10.1080/14427591.2022.2057574 (2023).

[6] Changala, M. & Ndhlovu, E. *Sport, Leisure and Recreation Preferences among Older Persons in Lusaka Urban District* (Implications for Adult Education Programmes, 2020).

[7] Craig, C. L. et al. International physical activity questionnaire: 12-country reliability and validity. *Med. Sci. Sports Exerc. ***35** (8), 1381–1395 (2003). 12900694 10.1249/01.MSS.0000078924.61453.FB

[8] Croezen, S., Avendano, M., Burdorf, A. & van Lenthe, F. J. Social participation and depression in old age: a fixed-effects analysis in 10 European countries. *Am. J. Epidemiol. ***182** (2), 168–176. 10.1093/aje/kwv015 (2015). 26025236 10.1093/aje/kwv015 PMC4493978

[9] Das, A. et al. A systematic review of loneliness and social isolation scales used in epidemics and pandemics. *Psychiatry Res. ***306**, 114217 (2021). 34644661 10.1016/j.psychres.2021.114217 PMC8502233

[10] Djukanovic, I., Carlsson, J. & Årestedt, K. Is the hospital anxiety and Depression Scale (HADS) a valid measure in a general population 65–80 years old? A psychometric evaluation study. *Health Qual. Life Outcomes*. **15**, 1–10 (2017). 28978356 10.1186/s12955-017-0759-9 PMC5628437

[11] Domènech-Abella, J. et al. Loneliness and depression in the elderly: the role of social network. *Soc. Psychiatry Psychiatr. Epidemiol. ***52** (4), 381–390. 10.1007/s00127-017-1339-3 (2017). 28154893 10.1007/s00127-017-1339-3

[12] Erzen, E. & Çikrikci, Ö. The effect of loneliness on depression: a meta-analysis. *Int. J. Soc. Psychiatry*. **64** (5), 427–435 (2018). 29792097 10.1177/0020764018776349

[13] Faronbi, G. O., Faronbi, J. O. & Adegbenro, C. A. Post Retirement Work Engagement among the Elderly in Ile Ife, Osun-State, Nigeria.

[14] Faronbi, G. O., Faronbi, J. O. & Adegbenro, C. A. Post-retirement economic work engagement among the elderly in Ile-Ife, Osun-state, Nigeria. Nigerian journal of nursing. *Nigerian J. Nurs.* (2021).

[15] Faronbi, J. O., Awoleye, T. E., Idowu, O. A. & Olagbegi, O. M. Association of nutrition, physical activity, and morbidity among older adults. *J. Public Health*. 10.1007/s10389-023-02186-8 (2024).

[16] Fiske, A., Wetherell, J. L. & Gatz, M. Depression in older adults. *Ann. Rev. Clin. Psychol. ***5**, 363–389 (2009). 19327033 10.1146/annurev.clinpsy.032408.153621 PMC2852580

[17] Giles-Corti, B. & Donovan, R. J. Socioeconomic status differences in recreational physical activity levels and real and Perceived Access to a supportive physical environment. *Prev. Med. ***35** (6), 601–611. 10.1006/pmed.2002.1115 (2002). 12460528 10.1006/pmed.2002.1115

[18] Goh, S. Y., Yoon, E. K. & Lee, M. N. Effectiveness of physical activity program in improving the self-esteem of female elderly in rural areas. *Asia-Pacific J. Convergent Res. Interchange FuCoS*. **7** (11), 151–163 (2021).

[19] Gonzales, E., Matz-Costa, C. & Morrow-Howell, N. Increasing opportunities for the Productive Engagement of older adults: a response to Population Aging. *Gerontologist*. **55** (2), 252–261. 10.1093/geront/gnu176 (2015). 26035601 10.1093/geront/gnu176

[20] Gustafsson, S., Berglund, H., Faronbi, J., Barenfeld, E. & Ottenvall Hammar, I. Minor positive effects of health-promoting senior meetings for older community-dwelling persons on loneliness, social network, and social support. *Clin. Interv. Aging*, 1867–1877. (2017). 10.2147/CIA.S143994 PMC5683788 29158669

[21] Harlow, R. E. & Cantor, N. Still participating after all these years: a study of life task participation in later life. *J. Personal. Soc. Psychol. ***71** (6), 1235 (1996). 10.1037//0022-3514.71.6.1235 8979389

[22] Hassan, S. S., Amin, N. M. & Mohamed, N. A. Relationship between loneliness and Depression among Elderly in Minia City. *Biomed. Nurs. ***3** (4). 10.7537/marsbnj030417.11 (2017).

[23] He, W., Kowal, P. & Naidoo, N. *Trends in Health and Well-Being of the Older Populations in SAGE Countries: 2014–2015*. (2018).

[24] Hughes, M. E., Waite, L. J., Hawkley, L. C. & Cacioppo, J. T. A short scale for measuring loneliness in large surveys: results from two population-based studies. *Res. Aging*. **26** (6), 655–672 (2004). 18504506 10.1177/0164027504268574 PMC2394670

[25] Igbokwe, C. C. et al. Prevalence of loneliness and association with depressive and anxiety symptoms among retirees in Northcentral Nigeria: a cross-sectional study. *BMC Geriatr. ***20**, 1–10 (2020). 10.1186/s12877-020-01561-4 PMC7178938 32326891

[26] Iloh, G. U. P., Aguocha, G. U., Amadi, A. N. & Chukwuonye, M. E. Depression among Ambulatory Adult patients in a primary care clinic in Southeastern Nigeria. *Nigerian Postgrad. Med. J. ***25** (4), 204–212. 10.4103/npmj.npmj_107_18 (2018). 10.4103/npmj.npmj_107_18 30588940

[27] Kadariya, S., Gautam, R. & Aro, A. R. Physical activity, mental health, and wellbeing among older adults in South and Southeast Asia: a scoping review. *BioMed Research International*, *2019*. (2019). 10.1155/2019/6752182 PMC6925721 31886239

[28] Kharicha, K., Manthorpe, J., Iliffe, S., Davies, N. & Walters, K. Strategies employed by older people to manage loneliness: systematic review of qualitative studies and model development. *Int. Psychogeriatr. ***30** (12), 1767–1781 (2018). 29798736 10.1017/S1041610218000339

[29] Krause-Parello, C. A., Gulick, E. E. & Basin, B. Loneliness, depression, and physical activity in older adults: the therapeutic role of human–animal interactions. *Anthrozoös*. **32** (2), 239–254 (2019).

[30] Lennartsson, C. & Silverstein, M. Does Engagement with Life Enhance Survival of Elderly people in Sweden? The role of Social and Leisure activities. *Journals Gerontology: Ser. B*. **56** (6), S335–S342. 10.1093/geronb/56.6.S335 (2001). 10.1093/geronb/56.6.s335 11682594

[31] Lin, Z. & Feng, Z. Influence of physical activity on self-esteem in elderly population narrative review. *Int. Neurourol. J. ***28** (1), 555–562 (2024).

[32] Miceli, S., Maniscalco, L. & Matranga, D. Social networks and social activities promote cognitive functioning in both concurrent and prospective time: evidence from the SHARE survey. *Eur. J. Ageing*. **16**, 145–154 (2019). 31139029 10.1007/s10433-018-0486-z PMC6509309

[33] Milanović, Z. et al. Age-related decrease in physical activity and functional fitness among elderly men and women. *Clin. Interv. Aging*, 549–556. (2013). 10.2147/CIA.S44112 PMC3665513 23723694

[34] Naing, L., Winn, T. & Rusli, B. Practical issues in calculating the sample size for prevalence studies. *Archives Orofac. Sci. ***1**, 9–14 (2006).

[35] National Institute on Ageing. Social isolation, loneliness in older people pose health risks. *Retrieved ***11**/03/2024, from (2019). https://www.nia.nih.gov/news/social-isolation-loneliness-older-people-pose-health-risks

[36] National Population Commission. *Nigeria National Census: Population Distribution by Sex, State, LGAs and Senatorial District: 2006 Census Priority Tables (Vol. 3).* Retrieved from (2006). http://www.population.gov.ng/index.php/publication/140-popn-distri-by-sex-state-jgas-and-senatorial-distr-2006

[37] Ojagbemi, A., Bello, T. & Gureje, O. The roles of depression and social relationships in the onset and course of loneliness amongst Nigerian elders. *Int. J. Geriatr. Psychiatry*. **36** (4), 547–557 (2021). 33091186 10.1002/gps.5451

[38] Ojagbemi, A. & Gureje, O. Social relationships and the association of loneliness with major depressive disorder in the Ibadan study of aging. *World Social Psychiatry*. **1** (1), 82–88 (2019).

[39] Oyeyemi, A. L., Oyeyemi, A. Y., Jidda, Z. A. & Babagana, F. Prevalence of physical activity among adults in a metropolitan Nigerian city: a cross-sectional study. *J. Epidemiol. ***23** (3), 169–177 (2013). 23604060 10.2188/jea.JE20120116 PMC3700262

[40] Paganini-Hill, A., Kawas, C. H. & Corrada, M. M. Activities and mortality in the elderly: the leisure world cohort study. *Journals Gerontol. Ser. A: Biomedical Sci. Med. Sci. ***66** (5), 559–567 (2011). 10.1093/gerona/glq237 PMC3074957 21350247

[41] Schrempft, S., Jackowska, M., Hamer, M. & Steptoe, A. Associations between social isolation, loneliness, and objective physical activity in older men and women. *BMC Public. Health*. **19**, 1–10 (2019). 30651092 10.1186/s12889-019-6424-y PMC6335852

[42] Sewdas, R. et al. Why older workers work beyond the retirement age: a qualitative study. *BMC Public. Health*. **17** (1), 672. 10.1186/s12889-017-4675-z (2017). 28830399 10.1186/s12889-017-4675-z PMC5567892

[43] Sheikh, J. I. & Yesavage, J. A. Geriatric Depression Scale (GDS): recent evidence and development of a shorter version. In Clinical Gerontology (165–173). Routledge. (2014).

[44] StataCorp. *Stata statistical software: Release 17: StataCorp LLC.*. (2023).

[45] Subair, O. et al. Relationship between family support and depression symptoms among older women attending a general practice clinic, Lautech Teaching Hospital, Osogbo, Nigeria. *Nigerian J. Family Pract. ***10** (3), 12–18 (2019).

[46] Taylor, W. D. Depression in the elderly. *N. Engl. J. Med. ***371** (13), 1228–1236 (2014). 25251617 10.1056/NEJMcp1402180

[47] Teixeira, C. M., Vasconcelos-Raposo, J., Fernandes, H. M. & Brustad, R. J. Physical activity, depression and anxiety among the elderly. *Soc. Indic. Res. ***113** (1), 307–318 (2013).

[48] Tiikkainen, P. & Heikkinen, R. L. Associations between loneliness, depressive symptoms and perceived togetherness in older people. *Aging Ment. Health*. **9** (6), 526–534. 10.1080/13607860500193138 (2005). 16214700 10.1080/13607860500193138

[49] Vaquera, E. 8 negative health effects of physical inactivity. *Retrieved ***11**/03/2024, from (2023). https://www.thehealthfeed.com/healthy-living/effects-of-physical-inactivity

[50] World Health, O. *Guidelines on Physical Activity, Sedentary Behaviour and Sleep for Children under 5 Years of age* (World Health Organization, 2019). https://iris.who.int/handle/10665/311664 31091057

[51] World Health Organization. Global strategy on diet, physical activity and health. (2018). https://iris.who.int/bitstream/handle/10665/43035/9241592222_eng.pdf?sequence=1

[52] World Health Organization. Social isolation and loneliness among older people: Advocacy brief. *Decade Healthy Aging Retrieved ***11**/03/2024, from (2021). https://www.who.int/publications/i/item/9789240030749

[53] World Health Organization. Physical activity. (2022). https://www.who.int/news-room/fact-sheets/detail/physical-activity

[54] Zimmer, Z. et al. Spirituality, religiosity, aging and health in global perspective: a review. *SSM Popul. Health*. **2**, 373–381. 10.1016/j.ssmph.2016.04.009 (2016). 29349154 10.1016/j.ssmph.2016.04.009 PMC5758000

